# Lipase‐catalyzed interesterification of Sunite sheep tail fat and flaxseed oil provides a fat having a unique fatty acid content and favorable physicochemical and nutritional properties

**DOI:** 10.1002/fsn3.4111

**Published:** 2024-03-24

**Authors:** Jin‐Hua Baoyindugurong, Jia‐Le Hou, Ya‐Nan Ren, Ya‐Wen Li, Mailisi Heshuote, Hugejiletu, Naoki Ohara, Yukiko Naito, Kenjiro Tatematsu

**Affiliations:** ^1^ College of Food Science and Engineering Inner Mongolia Agricultural University Hohhot China; ^2^ Inner Mongolia Key Laboratory of Biomanufacturing Hohhot China; ^3^ Institute of Nutrition Sciences Kagawa Nutrition University Sakado Saitama Japan; ^4^ School of Allied Health Sciences Kitasato University Sagamihara Kanagawa Japan; ^5^ Crystal Pharmatech, Inc. Cranbury New Jersey USA; ^6^ Columbia University New York New York USA; ^7^ Inner Mongolia Hesge Green Industry Import and Export Co., Ltd Xilingol China; ^8^ College of Pharmacy Kinjo Gakuin University Nagoya Aichi Japan; ^9^ Gifu Pharmaceutical University Gifu Japan

**Keywords:** cosmetics, flaxseed oil, healthy food, lipase‐catalyzed interesterification, sn‐2, Sunite sheep tail fat, α‐linolenic acid

## Abstract

The aim of this study is to combine flaxseed oil (FO), rich in α‐linolenic acid (ALA), with Sunite sheep tail fat (STF) through a lipase‐catalyzed transesterification reaction, in order to produce an edible oil with a fatty acid ratio suitable for human needs. Initially, the optimal conditions for esterification were determined using the Box–Behnken design, with the measurement criterion being the content of ALA at the sn‐2 position. The results indicated that the highest content of sn‐2 ALA was obtained under the conditions of using 6.8 wt% Lipozyme®RMIM as the catalyst, a reaction temperature of 57°C, a reaction time of 3.3 h, and a substrate mass ratio of 5.6:4.4 for STF and FO. This led to the rapid breaking and recombining of molecular bonds, resulting in the interesterified fat (IF) with the highest content of ALA at the sn‐2 position. Comparing STF and FO, IF exhibited excellent fatty acid composition and content. Furthermore, IF had a lower melting point and crystallization temperature compared to STF, and its solid fat content decreased with increasing temperature, completely melting at temperatures above 30°C. Thus, IF is a synthesized fat with excellent properties from both animal and vegetable sources.

## Graphical abstract

This new type of oil is rich in ALA and has excellent physical and chemical properties. Combined with the characteristics of Inner Mongolia and the international research results of transesterification, it will certainly attract the interest of scholars in the food industry.

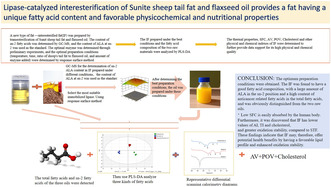

## 1 INTRODUCTION

Structured lipids (SLs) are new types of functional lipids produced by restructuring natural triglycerides by exchanging fatty acids (FAs). In addition to unique physicochemical characteristics, including melting point and oxidation stability, SLs possibly acquire favorable biological effects, for example, adjusting calorie intake, blood lipids, and immune function. Hence, the food industry is closely monitoring new SLs with great interest (Guo et al., 2020). Owing to the particular characteristics of lipase and phospholipase as biocatalysts, SLs have significant potential for commercial enzymatic production, as well (Kim & Akoh, 2015). Either chemical transesterification or lipase‐catalyzed interesterification may be used to synthesize SLs (Ma et al., 1999) and the lipase‐catalyzed one is the preferred method among these techniques. Lipase‐catalyzed interesterification, in comparison to chemical transesterification, offers several advantages, including milder reaction conditions, simpler postreaction procedures, greater controllability, and reduced impact of the production process on the quality and stability of the products. Additionally, this method helps to prevent the loss of flavor and nutritive value (Hee et al., 2008; Koçak et al., 2011; Soupas et al., 2005).

Functional products have been developed to leverage the easy absorbability of FAs located at the sn‐2 position of triglycerides. By introducing unsaturated FAs (UFAs) into this position, these products provide the advantages of consuming low‐calorie lipids (Iwasaki & Yamane, 2000). Additionally, through an enzymatic reaction, binding n‐3 polyunsaturated FAs (PUFAs) to the sn‐2 position of the glycerol backbone can provide enhanced protection against the oxidation of PUFAs (Wijesundera et al., 2008).

Due to their harsh living conditions, Sunite sheep rely on their valuable nutrient reserve, known as Sunite sheep tail fat (STF), to sustain them during periods of food shortages. According to traditional Mongolian medicine, STF is believed to enhance vital energy, known as “Yang” in Chinese medicine. It is also believed to contribute to improving body tone, skin moisturization, body warmth, and protection against pathogens, as well as enhancing kidney function (Tienan & Li, 2021). STF contains a distinctive composition of odd‐numbered chain‐saturated FAs (OCSFAs) that are exclusive to ruminant animals. Examples of these OCSFAs found in STF include pentadecanoic acid and heptadecanoic acid (Aksu, 2009). It has been reported that STF also contains branched‐chain FAs (BCFA) (Dan et al., 2019). BCFAs possess a low freezing point and exhibit favorable thermal and oxidation stability.

In recent years, there has been increasing concern about the imbalanced intake of UFAs. The World Health Organization (WHO) recommends a dietary intake ratio of 4:1 for linoleic acid (LA, n‐6) to α‐linolenic acid (ALA, n‐3). Similarly, the Chinese Nutrition Society suggests a recommended range of 4:1 to 6:1 for this ratio (Chinese Nutrition Society, 2022). Currently, the average ratio of n‐6 to n‐3 PUFAs in the Chinese population's diet is 8.6:1 or even higher (Jian et al., 2022). Due to the conversion of ALA into eicosapentaenoic acid (EPA) and docosahexaenoic acid, the supplementation of ALA has become an urgent concern in the field of public health and nutrition.

As noted above, the effect of STF on enhancing the vital energy known as “Yang,” which is associated with bolstering the body's disease resistance, may be attributed to its unique FA composition. However, it is important to note that STF, being an animal fat, contains significantly lower amounts of PUFAs compared to the majority of plant oils. Among plant oils that are rich in PUFAs, flaxseed oil (FO), perilla oil, and sacha inchi nut oil are particularly noteworthy. These oils contain a high concentration of ALA, with ALA comprising approximately half of the total FAs present in these oils. Given the current deficiency of ALA in diets, interesterified fat (IF) with its favorable FA profiles and physicochemical properties may offer several advantages as a health food and as a cosmetic ingredient. In the present study, enzymatic interesterification was conducted using STF and FO as the raw materials, resulting in the production of IF with distinctive FA compositions.

The raw materials used for enzymatic transesterification of oil are goat tail fat, which has not been deeply developed and utilized in Inner Mongolia, China, and flaxseed oil, which has been widely exported and applied. Through the preexperiment to determine the most suitable for the transesterification of the two lipases, and the use of this lipase to explore the best preparation conditions of binary oil. Process innovation, the use of rotary evaporation instrument to ensure that the grease is not oxidized in the transesterification process, compared with the traditional magnetic stirrer can better ensure that the immobilized lipase is not destroyed in the transesterification process, so that it is easier to separate from the product after the completion of transesterification.

## 2 MATERIALS AND METHODS

### 2.1 Materials and reagents

STF was prepared from Sunite sheep tails (Xilingol League, Inner Mongolia, China) using the patented method by Baoyindu Gulong Jin‐Hua (Jinhua et al., 2018). FO was purchased from Inner Mongolia Greeno Biological (Inner Mongolia, China). Lipozyme®TL IM, Lipozyme®CALB, Lipozyme®RM IM, and Novozyme®435 were from Novozymes Biotechnology (Beijing, China). Boron trifluoride methanol solution, n‐hexane, and acetone were of the highest commercially available purity grade.

### 2.2 Selection of lipase

The optimal lipase for the study was chosen from a selection of four enzymes, including nonspecific Lipozyme®CALB and Novozym®435, as well as sn‐1 and sn‐3 position‐specific immobilized enzymes, Lipozyme®TL IM and Lipozyme®RM IM. The interesterification process using STF and FO was conducted under the following conditions. The reaction temperatures were 65°C for Lipozyme®RM IM, 60°C for Novozyme®435 and Lipozyme®CALB, and 55°C for Lipozyme®TL IM, respectively. The reaction time was 3 h, using 4 wt% of one of the enzymes and a 6:4 mass ratio of STF to FO. The catalytic efficiencies of the enzymes were assessed based on the levels of ALA present at the sn‐2 position of the obtained triglycerides. The enzyme that yielded the highest ALA content was selected as the optimal enzyme for the study. The substrate mixture, along with each enzyme, was incubated in a water bath at the optimal temperature, while a rotary evaporator operating at 60 r/min facilitated vacuum extraction. Following the completion of the reaction, the lipase was separated by centrifugation at 7100 ×*g* for 3 min at 4°C. The analysis of ALA content at the sn‐2 position of the obtained interesterified triglycerides was performed using gas chromatography–mass spectrometry (GC–MS, AGILENT Technology Co., LTD).

### 2.3 Preexperiments were performed before analysis using the Box–Behnken design for response surface methodology

The interesterification reactions were carried out under the following 20 different conditions to determine the suitable ranges for reaction temperature, reaction time, amount of enzyme, and substrate mass ratio. These data were gathered for the purpose of the Box–Behnken design for response surface methodology (BBDRSM) analysis.

1. Reaction time, 3 h; temperature, 30, 40, 50, 60, and 70°C; concentration of Lipozyme®RM IM, 4 wt%; mass ratio of STF and FO, 6:4
2. Reaction time, 1, 2, 3, 4, and 5 h; temperature, 60°C; concentration of Lipozyme®RM IM, 4 wt%; mass ratio of STF and FO, 6:4
3. Reaction time, 3 h; temperature, 60°C; concentrations of Lipozyme®RM IM, 2, 4, 6, 8, and 10 wt%; mass ratio of STF to FO, 6:4
4. Reaction time, 3 h; temperature, 60°C; concentration of Lipozyme®RM IM, 4 w%; mass ratios of STF to FO, 5:5, 6:4, 7:3, 8:2, and 9:1

The catalytic efficiency of the enzyme was assessed based on ALA content at the sn‐2 position of the obtained triglycerides.

### 2.4 Selection of optimal reaction conditions using BBDSRM

The content of ALA at the sn‐2 position of the triglycerides in IF was used as the response value (an indicator) to evaluate the outcome of the reaction. The reaction conditions for the interesterification were assessed and determined using BBDSRM. That is, based on the results of the preliminary experiments, three successive values for each variable of the reaction conditions were assigned to −1, 0, and +1, respectively (Table 1). Then, the optimal reaction condition was determined using BBDRSM (Design Expert 11.1.0.1; Stat‐Ease, Minneapolis, MN, USA).

**TABLE 1:** Data allocation for BBDRSM analysis.

Independent variables	Horizontal code		
	−1	0	1
A: Reaction temperature (°C)	50	60	70
B: Reaction time (h)	2	3	4
C: Enzyme addition (wt%)	4	6	8
D: Substrate mass ratio (S:F)	5:5	6:4	7:3

*Note*: The Box–Behnken Design model was utilized to design the response surface experiment, in order to identify the optimal reaction condition. The content of α‐linolenic acid at the sn‐2 position in the interesterified fat was considered as the response value in the experiment. For details, refer to the text.

Abbreviation: STF:FO: the mass ratio of Sunite sheep tail fat to flaxseed oil.

### 2.5 Determination of FA contents in STF, FO, and IF

The oil and fat samples were methyl esterified. Three milliliters of 0.5 mol/L potassium hydroxide–methanol solution was mixed with 2 mL of the sample. The mixture was placed in boiling water for 30 min, followed by cooling to room temperature. The mixture was supplemented with 4 mL of a 14% solution of boron trifluoride in methanol and subjected to boiling water for 5 min. To extract FA methyl esters, 2 mL of n‐hexane and 5 mL of saturated saline were added to the reaction solution. The solution was thoroughly mixed and left to stand at room temperature for approximately 30 s. Subsequently, the supernatant was separated and utilized for GC–MS analysis.

The following GC–MS conditions were used. A Supelco®SPTM‐2560 gas capillary column with dimensions of 100 m × 0.25 mm × 0.2 μm was employed. The injection port and detector temperatures were set at 260°C. A constant column flow rate of 1.2 mL/min was maintained, with an injection volume of 2.0 μL and a split ratio of 50:1.

The column oven program involved an initial temperature of 100°C with a 5‐min holding time. Subsequently, the temperature was increased to 200°C at a rate of 4°C/min and held for 10 min. Finally, the temperature was further increased to 240°C at a rate of 1°C/min and maintained for 5 min.

For MS analysis, the ion source temperature was set at 230°C, while the quadrupole temperature was set at 150°C. Electron bombardment energy was set at 70 eV, and the scanning range for mass detection was from 30 to 400 *m*/*z*. The auxiliary temperature was maintained at 230°C.

### 2.6 Determination of FA contents at sn‐2 position of triglycerides

A 20‐mg aliquot of the sample was taken. It was then mixed with 2 mL of 1 mol/L Tris–HCl buffer (pH 8.0), 0.5 mL of 1 g/L sodium cholate solution, 0.2 mL of 22.2% CaCl_2_ solution, and 20 mg of pancreatic lipase and vortexed for 1 min at 40°C. One milliliter of 6 mol/L HCl solution and 2 mL of ether were added, and the resultant mixture was vortexed for 1 min. Subsequently, the mixture was centrifuged at 2500 ×*g* for 5 min at 4°C. One milliliter of the upper ether layer was taken and blown dry with nitrogen at 45°C. To dissolve the dried enzymolysis products obtained, 1 mL of n‐hexane was added to the mixture. Then, 1 mL of the enzymolysis product solution was loaded onto an activated amino column. The column was then eluted with 5 mL of 15 vol/% acetone‐n‐hexane solution, followed by an additional elution with 5 mL of 50 vol% acetone‐n‐hexane solution. The eluate was collected and dried under nitrogen at 45°C for GC–MS analysis.

### 2.7 Monitoring of melting point and crystallization temperature

A differential scanning calorimeter (Perkin Elmer Instruments, MA, USA) was used for monitoring the melting and crystallizing temperatures of fat and oil. A 5‐ to 10‐mg aliquot of oil or fat was placed in an aluminum box and heated from 25 to 60°C at a rate of 10°C/min, and the temperature was held for 5 min. Then, the sample was cooled to −60°C at a rate of −3°C/min, and the temperature was held for 5 min. Finally, the sample was heated again to 60°C at a rate of 3°C/min. The data obtained were analyzed by TA Universal Analysis 2000 thermal analysis software (Informer Technologies, https://www.informer.com/). SFC in FO was not measured, since FO is liquid in the temperature range examined.

### 2.8 Atherogenic and thrombogenic indices

The atherogenic index (AI) and thrombogenic index (TI) of the sample were calculated by substituting the percentage of FAs into the following formulas (Ulbricht & Southgate, 1991):

$$\mathrm{AI}=\left(\text{lauric acid}+4\times \text{myristic acid}+\text{palmitic acid}\right)/\left(\text{∑MUFA}+\mathrm{\sum n}6\mathrm{FA}+\mathrm{\sum n}3\mathrm{FA}\right)$$

$$\mathrm{TI}=\left(\text{myristic acid}+\text{palmitic acid}+\text{stearic acid}\right)/\left(0.5\times \text{∑MUFA}+0.5\times \mathrm{\sum n}6\mathrm{FA}+3\times \mathrm{\sum n}3\mathrm{FA}\right)$$

where MUFA is the monounsaturated fatty acid; n3FA is the n‐3 fatty acid; and n6FA is the n‐6 fatty acid.

### 2.9 Measurement of AV, PV, and SFC of IF

The methods in Chinese National Standards GB 5009.229‐2016, GB 5009.2270‐201, and HLC (column, C18 reversed‐phase column of 4.6 mm × 150 mm, 3 μm; column temperature, 38°C; mobile phase, methanol; flow rate, 1.0 mL/min; measurement wavelength, 205 nm; injection volume, 10 μL) in GB 5009.128‐2016 and B/T 31743‐2015 were used to determine the acid value (AV), peroxide value (PV), cholesterol, and solid fat content (SFC) of the fat and oil, separately.

### 2.10 Data analysis and statistical methods

**FIGURE 1:**
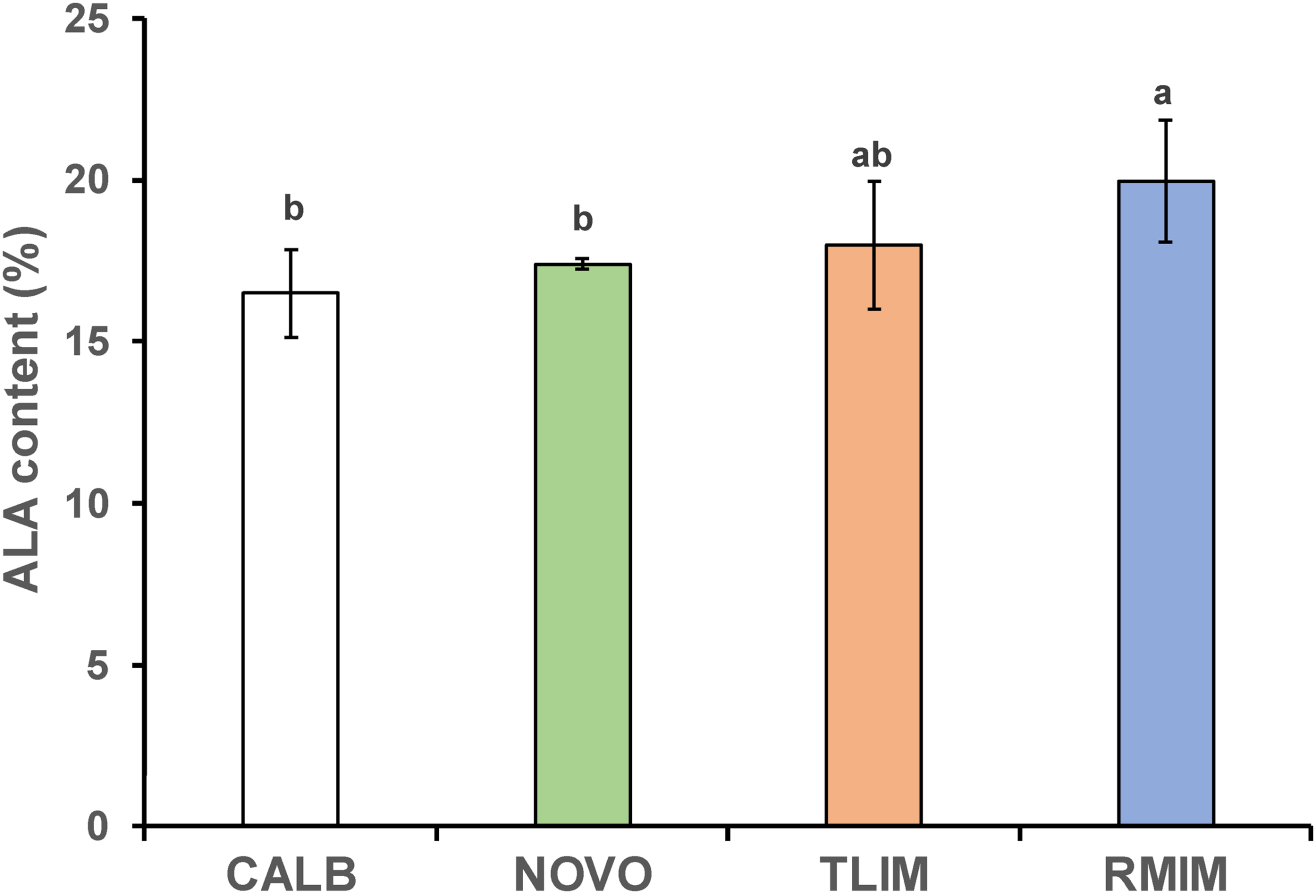
ALA contents at the sn‐2 position of triglycerides in the interesterified fats obtained using four lipases. ALA content in the interesterification product using Lipozyme®RM IM was significantly greater than when using Novozym®435 or Lipozyme®CALB. The reaction time, 3 h; the amount of an enzyme added, 4 wt%; the mass ratio of Sunite sheep fat to flaxseed oil, 6:4; the reaction temperatures, 60°C for CALB, 65°C for RMIM, 55°C for TLIM, and 60°C for NOVO. ALA, α‐linolenic acid; CALB, Lipozyme®CALB; NOVO, Novozyme®435; TLIM, Lipozyme®TL IM; RMIM, Lipozyme®RM IM. Values are means ± SDs of five experiments. The same alphabetical labels indicate the absence of significant differences between the groups (*p* > .05, Turkey's test).

The results are presented as means±SDs in Tables 4 and 5 (except for AI and TI), Figures 1, 4a, and 6. Excel 2021 software (Microsoft, Redmond, WA, USA) was used to collate and calculate the data. The differences between group mean values were evaluated by one‐way ANOVA followed by Turkey's test using SPSS Statistics 25.0 software (IBM, Armonk, New York (NY), USA) and considered to be significant when *p* < .05.

BBDRSM in the Design Expert 11.1.0.1 software (State‐East, NY, NY, USA) was used to design the response surface test and analyze the variance of the model. The partial least squares discriminant analysis (PLS‐DA) module of SIMCA® 14.1 software (MKS Data Analytics Solutions, Norecopa, Ås, Norway) and Origin 2021 software (OriginLab, Northampton, MA, USA) were used for PLS‐DA, calculating the variable importance in the projection (VIP) of forecast variables (the contribution rate of various FAs in the model established), and drawing related charts, respectively.

## 3 RESULTS

### 3.1 Lipase selection by catalytic efficiencies in interesterification reaction

As shown in Figure 1, ALA contents at the sn‐2 position of triglyceride molecules of IF obtained were 19.95%, 17.97%, 17.40%, and 16.50% for Lipozyme®RM IM, Lipozyme®TL IM, Novozym®435, and Lipozyme®CALB, respectively. ALA content in the interesterification product using Lipozyme®RM IM was significantly greater than when using Novozym®435 or Lipozyme®CALB. The reaction time, 3 h, the amount of an enzyme added, 4 wt%, the ALA initial content of mixture (the mass ratio of Sunite sheep fat to flaxseed oil, 6:4); the reaction temperatures, 60°C for CALB, 65°C for RMIM, 55°C for TLIM, and 60°C for NOVO.ALA, a‐linolenic acid.

Catalytic efficiency of an sn‐1 and sn‐3 specific lipase Lipozyme®RM IM was significantly stronger than that in the two other, nonspecific ones and tended to be stronger than the sn‐1 and sn‐3 specific one, Lipozyme®TL IM. Therefore, Lipozyme®RM IM was selected and used for the subsequent experiments.

### 3.2 The change of ALA content in sn‐2 position of IF in previous experiment

**FIGURE 2:**
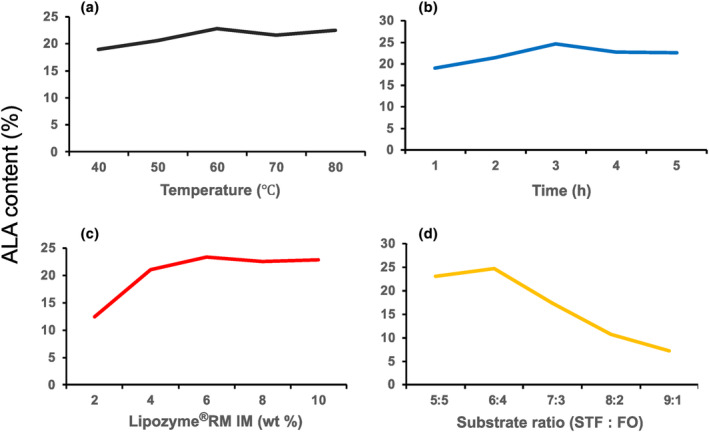
Influences of single factors on ALA contents in IF. Changes in ALA content at the sn‐2 position of triglycerides in the interesterified fats obtained by the reaction for 3 h at 30, 40, 50, 60, and 70°C using 4 wt% Lipozyme®RM IM with a 6:4 mass ratio of STF to FO (a); by the reaction for 1, 2, 3, 4, and 5 h at 60°C, using 4 wt% Lipozyme®RM IM with a 6:4 mass ratio of STF to FO (b); for 3 h at 60°C using 2, 4, 6, 8, and 10 wt% Lipozyme®RM IM with a 6:4 mass ratio of STF to FO (c); for 3 h at 60°C using 4 wt/% Lipozyme®RM IM with 5:5, 6:4, 7:3, 8:2, or 9:1 mass ratios of STF to FO (d) are shown. ALA, α‐linolenic acid; STF:FO, the mass ratio of Sunite sheep tail fat to flaxseed oil. Values are the means of three experiments.

Based on the results shown in Figure 2, the appropriate reaction conditions were determined as follows: a reaction temperature of 60°C, a reaction time of 3 h, an enzyme concentration of 6 wt%, and a substrate ratio of 6:4. Changes in ALA content at the sn‐2 position of triglycerides in the interesterified fats obtained by the reaction for 3 h at 30, 40, 50, 60, and 70°C using 4 wt% Lipozyme®RM IM with a 6:4 mass ratio of STF to FO (A); by the reaction for 1, 2, 3, 4, and 5 h at 60°C, using 4 wt% Lipozyme®RM IM with a 6:4 mass ratio of STF to FO (B); for 3 h at 60°C using 2, 4, 6, 8, and 10 wt% Lipozyme®RM IM with a 6:4 mass ratio of STF to FO (C); for 3 h at 60°C using 4 wt/% Lipozyme®RM IM with 5:5, 6:4, 7:3, 8:2, or 9:1 mass ratios of STF to FO (D) are shown.

The results showed that the coefficient of determination (*R*; Kim & Akoh, 2015) was 0.9877, *p* < .0001, mismatch was not significant (*p* = .8792), and the correction coefficient (Adj *R*; Kim & Akoh, 2015) was 0.9754, it is shown that the model can explain 97.54% of the variation of response values. These results indicated that the model was extremely significant, the uncertain factors had little effect on the interesterification reaction, and the fitting degree was good. The model can explain the relationship between the response value and each factor, and the influence of each factor on the test results.

### 3.3 Results of BBDRSM analysis

**FIGURE 3:**
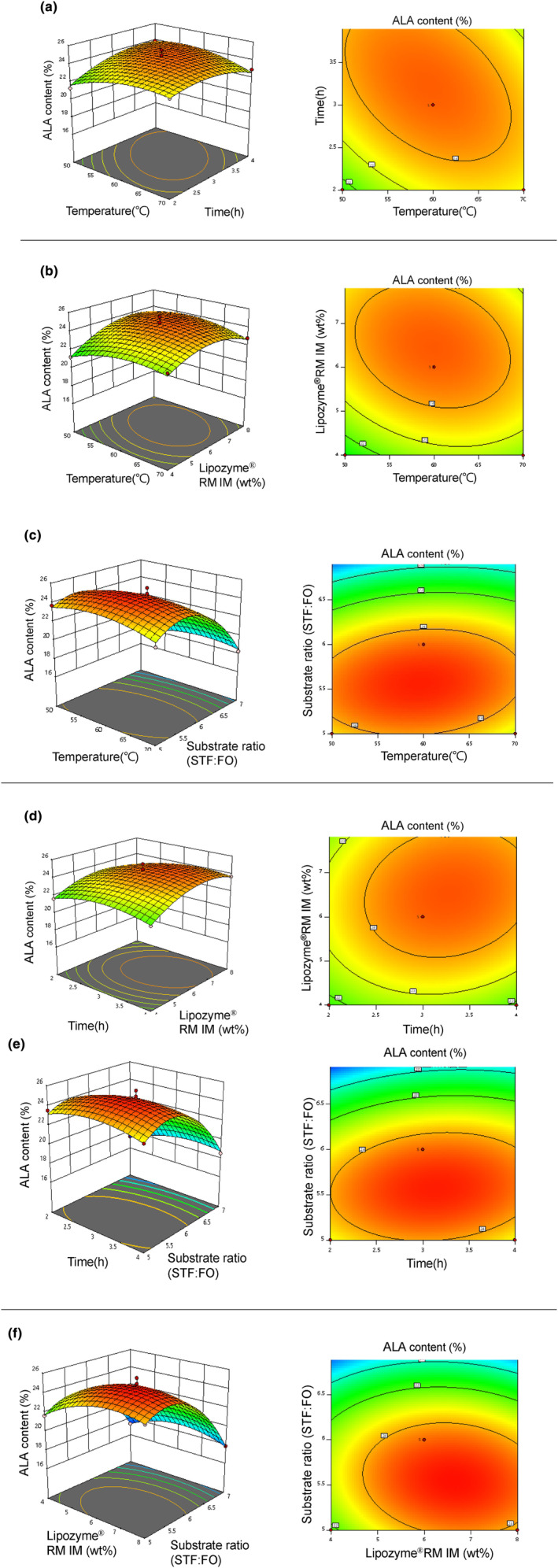
Response surfaces and contour plots BBDRSM. The influence of the interactions between the factors on the content of ALA at sn‐2 position of triglycerides in IF was analyzed by the 3D response surfaces (left) and contour plots (right) using Design‐Expert software. Influence of the reaction temperature and time (a), the reaction temperature and amount of Lipozyme®RM IM (b), the reaction temperature and STF:FO (c), the reaction time and the amount of Lipozyme®RM IM (d), the reaction time and STF:FO (e), and the amount of Lipozyme®RM IM and STS:FO (f). ALA, α‐linolenic acid; BBDRSM, Box–Behnken design for response surface methodology; FO, flaxseed oil; STF, Sunite sheep tail fat.

The data in Table 2 were used for the fitting by Design Expert 11.1.0.1 software of a second‐order regression model for the response surface. The values of *Y* = 24.54 + 0.0842A + 0.03883B + 0.6242C − 2.58D − 0.7175AB − 0.4575AC + 0.3975AD + 0.3050BC + 0.3775BD − 0.3900CD − 0.8471A (Kim & Akoh, 2015)–0.8633B^2^ − 1.30C^2^ − 3.90D^2^ were used to determine the optimal conditions for the interesterification reaction. The results of ANOVA for the regression by BBDRSM are shown in Table 3. It is shown that the coefficient of determination (*R*; Kim & Akoh, 2015) was 0.9877, *p* < .0001, mismatch was not significant (*p* = .8792), and the correction coefficient (Adj *R*; Kim & Akoh, 2015) was 0.9754, showing that the model explains 97.54% of the variability of response values. The model exhibited great significance, and the degree of fit was excellent. The response surfaces and the contour maps for all combinations of the factors are shown in Figure 3. The response surfaces between two of the four factors, which were allocated using a round‐robin technique, exhibited curvature. This indicates that the relationship between these factors and the response variable (content of ALA at the sn‐2 position of IF) is not linear. The curvature suggests that there is an interaction between these factors and that their combined effect influences the response variable in a nonlinear manner. In the contour maps drawn based on the curvature, the oval red region represents the maximum comfort zone. It was observed that the STF:FO mass ratio had the most significant influence on the Y value, the content of ALA at the sn‐2 position of IF. The factors were ranked in terms of their influence on the Y value and ranked as STF:FO > amount of Lipozyme®RM IM > reaction time > reaction temperature.

**TABLE 2:** ALA contents obtained in the preliminary experiments.

Trial number	A (h)	B (°C)	C enzyme addition (wt/%)	D substrate ratio (S:T)	ALA content
1	4	60	8	6:4	23.68
2	3	50	6	7:3	17.90
3	3	60	4	7:3	17.31
4	4	60	4	6:4	21.76
5	4	70	6	6:4	22.91
6	2	60	6	7:3	17.35
7	3	60	6	6:4	24.71
8	4	50	6	6:4	23.86
9	3	60	8	7:3	17.85
10	3	50	4	6:4	21.20
11	3	60	6	6:4	24.16
12	2	60	8	6:4	22.31
13	3	60	6	6:4	24.13
14	3	60	6	6:4	25.31
15	3	70	8	6:4	22.79
16	3	60	8	5:5	23.74
17	3	60	4	5:5	21.64
18	3	70	6	5:5	22.43
19	2	60	4	6:4	21.64
20	3	50	8	6:4	23.23
21	2	70	6	6:4	23.20
22	3	70	6	7:3	18.24
23	4	60	6	5:5	23.18
24	3	50	6	5:5	23.68
25	4	60	6	7:3	18.53
26	2	60	6	5:5	23.51
27	3	70	4	6:4	22.59
28	3	60	6	6:4	24.39
29	2	50	6	6:4	21.28

*Note*: ALA contents at the sn‐2 position of triglycerides in the interesterified fats obtained in the preliminary experiments were used as the response values in Box–Behnken design for response surface methodology analysis for finding the optimal reaction conditions.

Abbreviations: ALA, α‐linolenic acid; STF:FO, Sunite sheep tail fat:flaxseed oil.

**TABLE 3:** ANOVA table for the regression by BBDRSM analysis.

Source of variance	Sum of squares	Degrees of freedom	Mean square	*F*‐value	*p*‐Value
Model	157.41	14	11.24	80.33	<.0001
A – Temperature	0.085	1	0.085	0.6073	.4488
B – Time	1.81	1	1.81	12.93	.0029
C – enzyme addition	4.68	1	4.68	33.4	<.0001
D‐Scale	80.08	1	80.08	572.15	<.0001
AB	2.06	1	2.06	14.71	.0018
AC	0.8372	1	0.8372	5.98	.0283
AD	0.632	1	0.632	4.52	.0519
BC	0.3721	1	0.3721	2.66	.1253
BD	0.57	1	0.57	4.07	.0632
CD	0.6084	1	0.6084	4.35	.0559
A^2^	4.65	1	4.65	33.25	<.0001
B^2^	4.83	1	4.83	34.54	<.0001
C^2^	10.91	1	10.91	77.97	<.0001
*D* ^2^	61.97	1	61.97	442.72	<.0001
Residuals	1.96	14	0.14		
Mismatch	1	10	0.1003	0.4192	.8792
Pure error	0.9568	4	0.2392		
Sum	159.37	28			
*R* ^2^	.9877				<.0001
Adj *R* ^2^	.9754				

Abbreviations: BBDRSM, Box–Behnken design for response surface methodology; FO, flaxseed oil; STF, Sunite sheep tail fat.

### 3.4 Optimal interesterification condition

By using the regression equation model, the following optimal reaction conditions for the interesterification process were determined: the reaction temperature, 57.03°C; the reaction time, 3.32 h; the enzyme addition, 6.79 wt%; the substrate mass ratio, 5.56:4.44. These conditions were predicted to yield the desired outcome based on the regression model and were considered to be the optimal combination for achieving the desired ALA content at the sn‐2 position of IF. Under these conditions, the expected value of the content of ALA at the sn‐2 position was 25.27%. For the sake of convenience and practicality in the experimental procedures, the following reaction conditions were fixed: the reaction temperature was 57°C; the reaction time, 3.3 h; the enzyme addition, 6.8%; and the substrate mass ratio, 5.6:4.4.

Table 4 shows FA contents in STF, FO, and IF obtained through interesterification under optimal reaction conditions. In IF, the content of each FA tended to approach the average value found in ST and FO. In the interesterification process, most FA molecules did not show significant deflections in their exchange among the three materials. However, it was observed that certain FAs, specifically undecanoic acid, lauric acid, tridecanoic acid, and behenic acid, exhibited an increase in IF compared to their levels in STF and FO. These particular FAs experienced a notable change during the reaction, leading to their higher concentration in IF. The SFA:MFA:PUFA ratio in IF reached 1:1.2:1.1.

**TABLE 4:** Contents (%) of FAs and those bound at the sn‐2 of triglycerides in STF, FO, and IF.

FA		STF		FO		IO	
		Total	sn‐2	Total	sn‐2	Total	sn‐2
SFA							
c8:0	Linolenic acid	0.01 ± 0a	–	0 ± 0b	–	0.01 ± 0a	–
c10:0	Capric acid	0.11 ± 0.01a	–	0.008 ± 0b	–	0.09 ± 0.03a	–
c11:0	Undecanoic acid	–	–	–	–	0.02 ± 0	–
c12:0	Lauric acid	0.1 ± 0.01b	–	0.01 ± 0c	–	0.13 ± 0a	–
c13:0	Tridecanoic acid	0.02 ± 0a	–	0.01 ± 0b	–	0.02 ± 0.01a	–
c14:0	Myristic acid	2.79 ± 0.22a	3.11 ± 0.00A	0.05 ± 0.01c	0.67 ± 0.00C	2.28 ± 0.1b	1.69 ± 0.01B
c15:0	Pentadecanoic acid	0.48 ± 0.02a	–	0.02 ± 0c	–	0.3 ± 0.05b	–
c16:0	Palmitic acid	22.76 ± 0.39a	25.39 ± 0.12A	6.07 ± 0.03b	6.01 ± 0.01B	17.95 ± 0.15a	17.01 ± 0.01B
c17:0	Margaric acid	2.7 ± 0.13a	–	0.05 ± 0.02c	–	1.08 ± 0.05b	–
c18:0	Stearic acid	16.33 ± 1.22a	17.25 ± 0.01A	4.39 ± 0.03c	4.50 ± 0.00C	8.95 ± 0.15b	8.46 ± 0.01B
c20:0	Arachidic acid	0.2 ± 0.04a	–	0.09 ± 0.05b	–	0.11 ± 0.01b	–
c21:0	Heneicosanoic acid	0.41 ± 0.05	–	–	–	0.39 ± 0.04	–
c22:0	Behenic acid	0.03 ± 0.01b	–	0.03 ± 0.01b	–	0.05 ± 0a	–
c23:0	Tricosanoic acid	0.08 ± 0.01	–	–	–	–	–
MUFA							
c14:1	Myristoleic acid	0.16 ± 0.14	–	–	–	0.13 ± 0.01	–
c16:1	Palmitoleic acid	1.25 ± 0.73a	1.41 ± 0.00A	0.06 ± 0.01b	0.05 ± 0.00B	1.41 ± 0.9a	1.59 ± 0.00A
c17:1	Margaroleic acid	1.62 ± 0.06a	–	0.03 ± 0.01c	–	0.85 ± 0.01b	–
c18:1n9t	Trans‐Elaidic acid	3.87 ± 0.47a	–	0.03 ± 0b		0.07 ± 0.01b	–
c18:1	Oleic acid	44.01 ± 1.8a	48.74 ± 0.01A	17.43 ± 0.38c	17.49 ± 0.01C	33.98 ± 1.34b	37.27 ± 0.02B
c20:1	cis‐11‐Eicosenoicacid	0.14 ± 0.02b	–	0.17 ± 0.02a	–	0.13 ± 0.01b	–
PUFA							
c18:2n6c	LA	2.22 ± 0.24c	3.71 ± 0.01C	16.34 ± 0.23a	16.49 ± 0.04A	7. 92 ± 0.08b	8.29 ± 0.01B
c18:3n6	γ‐Linolenic acid	0.02 ± 0.01a	–	–	–	0.09 ± 0.01b	–
c18:3n3	ALA	0.26 ± 0.02c	0.37 ± 0.00C	54.6 ± 0.76a	54.64 ± 0.13A	23.84 ± 0.65b	24.71 ± 0.07B
c20:2	Eicosadienoic acid	0.07 ± 0.03a		0.02 ± 0b		0.04 ± 0.01b	
c20:3n6	cis‐8,11,14‐Eicosatrienoic acid	0.01 ± 0.01b	–	0.13 ± 0a	–	0.03 ± 0.01b	–
c20:3n3	cis‐11,14,17‐Eicosatrienoic acid	0.02 ± 0.01	–	–	–	0.02 ± 0.01	–
c20:4n6	Arachidonic acid	0.13 ± 0.01a	–	0.07 ± 0.02b	–	0.06 ± 0.03b	–
c20:5	EPA	0.09 ± 0.03a	–	0.08 ± 0a	–	0.04 ± 0.01b	–
∑ SFA		46.15 ± 2.12a	45.75 ± 1.11A	10.73 ± 0.12c	11.19 ± 0.18C	31.4 ± 0.39b	27.16 ± 2.3B
∑ MUFA		51.05 ± 2.06a	50.16 ± 1.22A	19.48 ± 2.34c	17.54 ± 0.21C	36.57 ± 0.78b	38.86 ± 1.49B
∑ PUFA		2.83 ± 0.19c	4.11 ± 0.19C	69.79 ± 2.34a	71.28 ± 0.24A	32.03 ± 0.61b	33.98 ± 1.19B
SFA:MFA:PUFA		16.3:18:1	11.1:12.2:1	1:1.8:6.5	1:1.6:6.4	1:1.2:1.1	1:1.4:1.3

*Note*: Total, total fatty acid contents; sn‐2, fatty acid contents in those bound at the sn‐2 of triglycerides. Values are means±SDs of 3 experiments.

Abbreviations: FA, fatty acid; FO, flaxseed oil; IF, interesterified fat; MUFA, mono‐unsaturated fatty acids; PUFA, polyunsaturated fatty acids; SFA, saturated fatty acids; STF, Sunite sheep tail fat.

**FIGURE 4:**
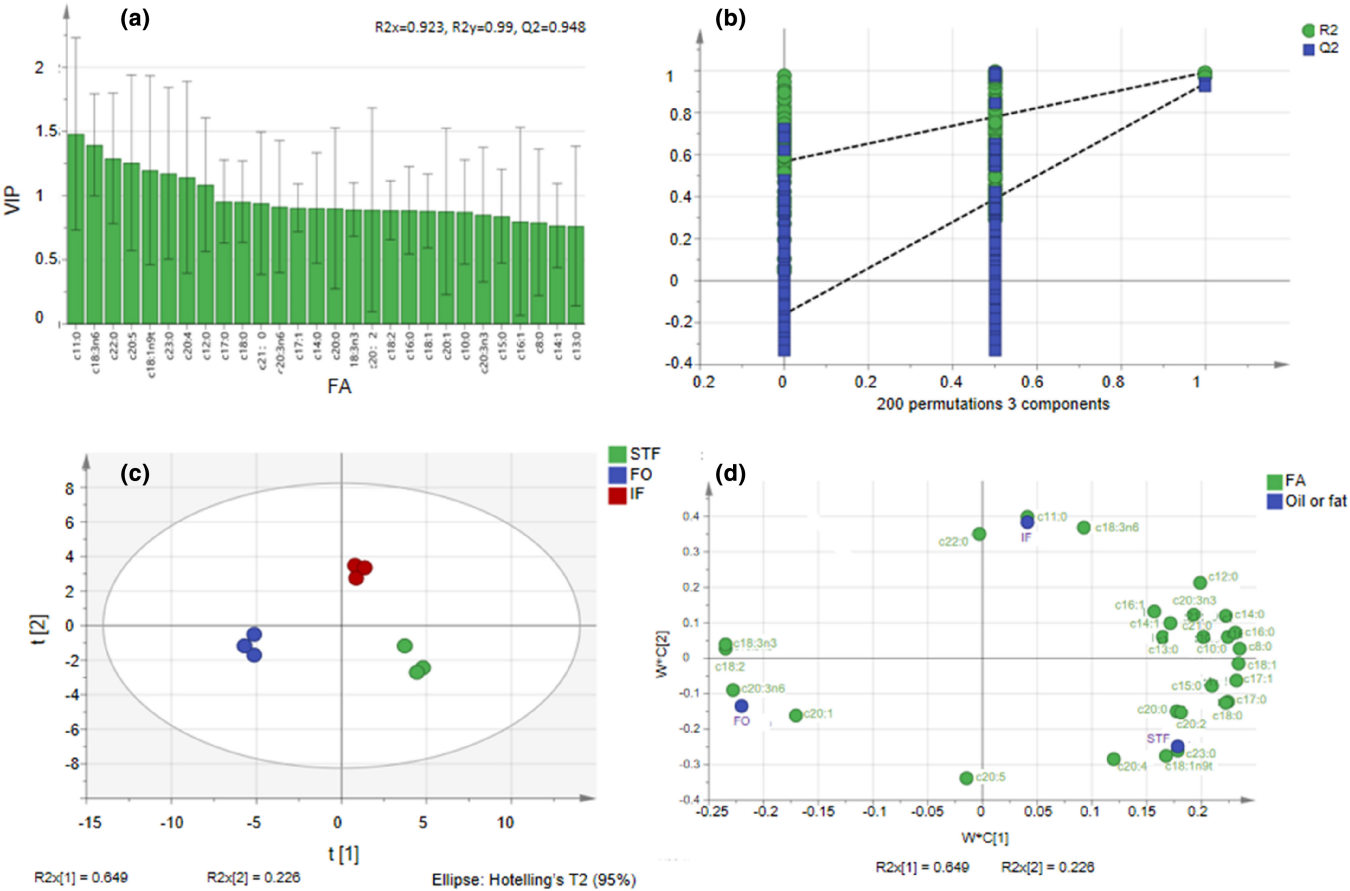
PLS‐DA. (a) The contribution of FAs in characterizing STF, FO, and IF. FAs with VIP values greater than 1 are considered significant in differentiating between STF, FO, and IF, and have a substantial impact on establishing a PLS‐DA model. (b) The predictive power of the model. *R*‐squared (R2, coefficient of determination) was used to evaluate the performance of a model in explaining the variation in the input variables and the output variables explained by the model. An R2 value close to 1 indicates a better fit of the model to the data. *Q*‐squared (Q2, predictive ability) represents the predictive ability. A Q2 value close to 1 indicates a high predictive ability of the model. The frequency of accuracy in 200 models in 200 replacement tests is plotted on the vertical axis and indicates the position of the PLS‐DA model accuracy. (c) PLS‐DA score chart for STF, FO, and IF. FA content of each oil or fat was measured three times. The abscissa shows the score value of the main component in the orthogonal signal correction (OSC) process, indicating the difference between group values. The ordinate shows the score value of the main component in the OSC process, the difference among three values within groups. (d) PLS‐DA root vector diagram for STF, FO, and IF. Analysis of the correlation between the oils/fats and specific FAs helps to identify the characteristic FA profiles of STF, FO, or IF for each and understand the changes that occur during interesterification. FA, fatty acid; FO, flaxseed oil; IF, interesterified fat; PLS‐DA, partial least squares discriminant analysis; STF, Sunite sheep tail fat; VIP, variable importance projection.

Undecanoic acid, behenic acid, ALA, and EPA contributed greatly to identifying IF. From the results in Table 4 and Figure 4a, it is shown that IF contained OCSFAs: undecanoic acid, tridecanoic acid, pentadecanoic acid, heptadecanoic acid, and heneicosanoic acid. In IF, the levels of these OCSFAs were higher than the average levels in both STF and FO, with the exception of heptadecanoic acid. This indicates that the interesterification process led to an increase in the levels of these OCSFAs in IF compared to the individual fats. These FAs, characterized by higher VIP values, are considered significant in classifying and discriminating between models for different oils and fats.

In Figure 4b, the fact that the intersection of the Q2 regression line with the vertical axis was at a point <0 after testing with 200 permutations is an indication that there is no overfitting in the model. The negative intersection point suggests that the model's predictive ability is better than random chance, providing evidence of its validity. Based on the results and analysis presented, it is reasonable to believe that the results obtained can be utilized for the separation and identification of oils. The identified characteristic FAs and their profiles, along with the established models and statistical analysis, provide valuable information for distinguishing and differentiating between different types of oils and fats. Overall, the identification of IF can be considered valid and reliable based on the provided experimental procedures, analytical techniques, and statistical analysis. The independent variable fitting index (R2x) was 0.923, the dependent variable fitting index (R2y) was 0.990, and the model prediction index (Q2) was 0.948. Thus, the model fitting results are considered acceptable, as R2 and Q2 exceed 0.5 (Yun et al., 2021). There were significant differences in FA composition between STF, FO, and IF; but there were no differences in the FA compositions of the three results for each, STF, FO, and IF. The esterification reaction had been successful (Figure 4c). Based on the plot in Figure 4d, the characteristic FAs of STF included tricosanoic acid (C23:0), eicosadienoic acid (C20:2), trans‐elaidic acid (C18:1n‐9t), arachidonic acid (C20:4n‐6), and stearic acid (C18:0). The characteristic FAs of FO included α‐linolenic acid (C18:3n‐3), linoleic acid (C18:2n‐6), cis‐8,11,14‐eicosatrienoic acid (C20:3n‐6), and cis‐11‐eicosenoic acid (C20:1). Lastly, the characteristic FAs of IF included behenic acid (C22:0), undecanoic acid (C11:0), and α‐linolenic acid (C18:3n‐6).

### 3.5 Analysis of the thermal properties of fat and oil

The thermal characteristics of specific fats and oils are influenced by the quantity, type, and positioning of FAs in triglyceride molecules. As the diversity of FAs bound to the molecules increases, the melting and crystallization curves exhibit multiple peaks. The primary crystalline forms observed are α, β′, and β (Chai et al., 2018).

**FIGURE 5:**
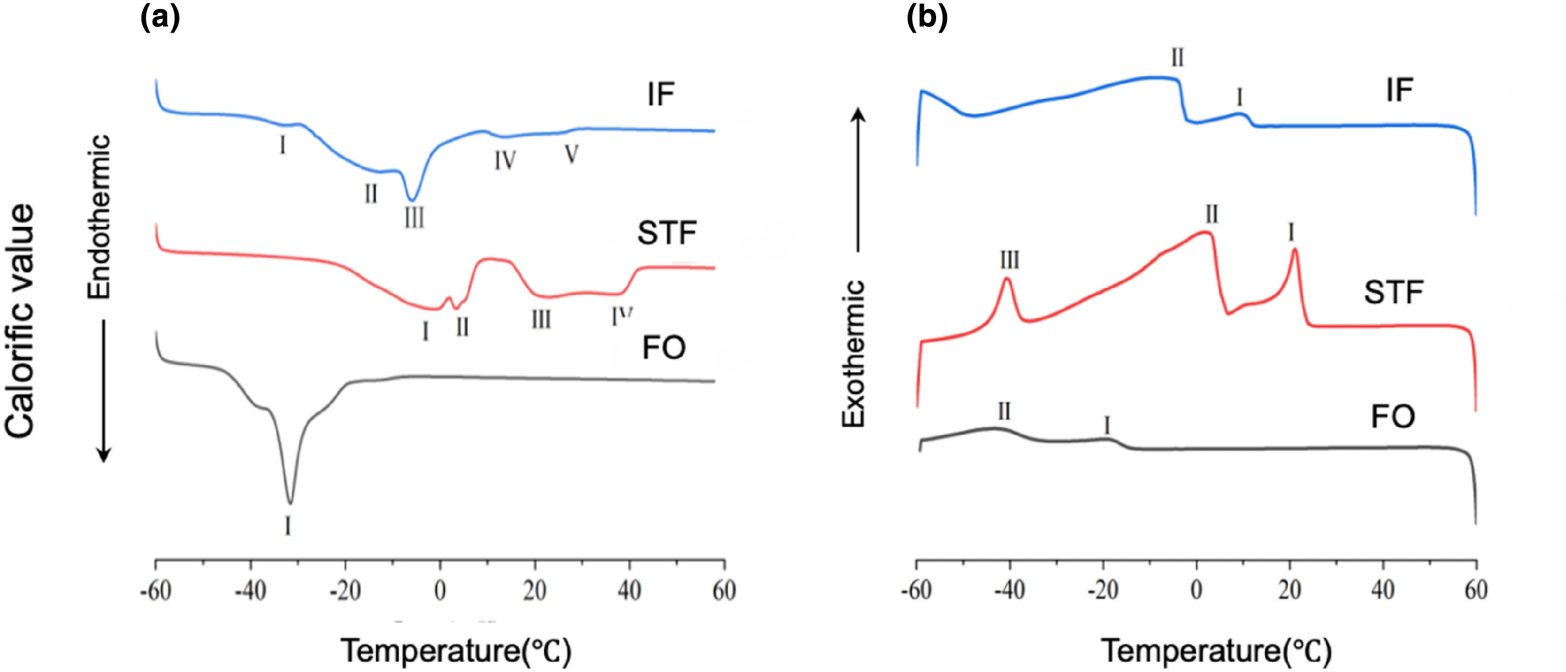
Representative differential scanning calorimetry diagrams of STF, FO, and IF. (a) Melting curves. (b) Crystallization curves. FO, flaxseed oil; IF, interesterified fat; STF, Sunite sheep tail fat.

In the melting curves, STF exhibited four endothermic, downward peaks at −0.17, 3.67, 20.41, and 37.61°C. FO exhibited a single peak at −31.76°C. IF exhibited five peaks, at −32.62, −13.83, −5.54, 13.19, and 26.16°C (Figure 5a). In the crystallization curves, STF exhibited three exothermic, upward peaks at 21.18, 2.91, and −40.40°C. FO exhibited two peaks at −19.51 and −41.93°C. IF also exhibited two peaks, at 9.33 and −7.50°C (Figure 5b). Due to the decrease in the content of saturated triglycerides in IF compared to that in STF, the peaks in the melting curves shifted toward the lower temperature range. Consequently, the melting began at a lower temperature. Additionally, in the melting curves of IF, peaks IV and V were smaller in magnitude than peaks II and III, and the amount of heat required for melting gradually decreased from peak III to peak V. The thermal properties of IF contribute to a refreshing melting sensation and a smooth texture. These properties are considered favorable and suitable for various applications in foods and cosmetics (Palla et al., 2017). Two β′ crystal forms were detected in STF, but not in IF. Peak IV in STF and peak V in IF were 37.61 and 26.16°C, respectively. The melting points obtained were 35.27 and 26.93°C for STF and IF, respectively.

**FIGURE 6:**
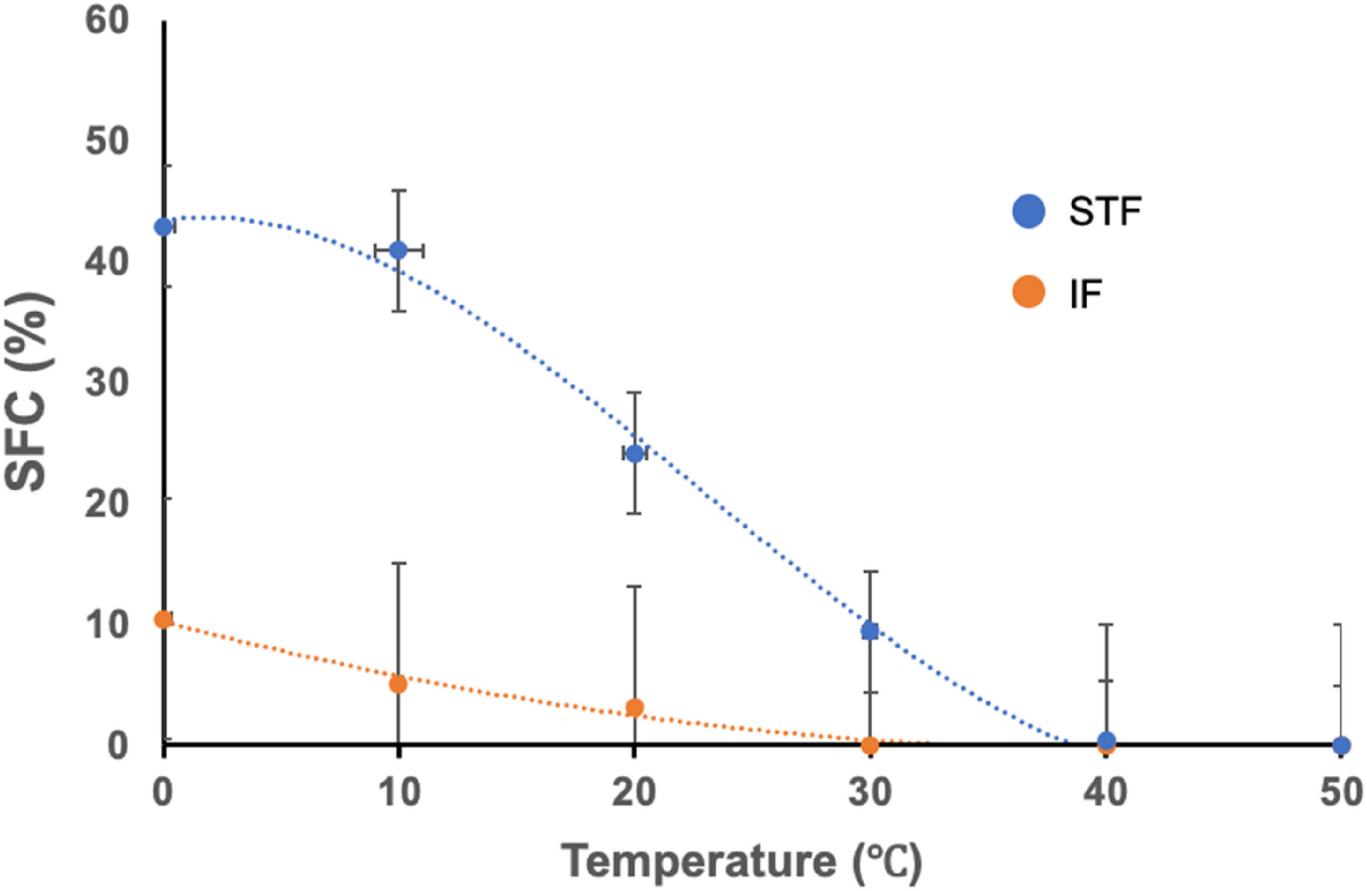
SFC of STF and IF. IF, interesterified fat; SFC, solid fat content; STF, Sunite sheep tail fat. Values are means ± SDs of three experiments.

SFC of STF consistently remained higher than that of IF within the temperature range of 0–30°C. As the temperature increased, SFC decreased in both STF and IF. SFC reached 0 at 30°C in IF and at approximately 40°C in STF (Figure 6). FO does not contain SFC.

### 3.6 AI, TI, and quality of STF, FO, and IF

AI, TI, cholesterol content, AV, and POV in STF, FO, and IF are shown in Table 5. AI and TI values in IF fell between the corresponding values observed in FO and STF. AV values were below 1 mg KOH/g and POV values were below 5.13 mmol/kg. Both values were in accordance with the industry standards outlined in GB15196‐2015. Cholesterol content in IF was slightly less than that in STF while the decrease was statistically significant.

**TABLE 5:** AI, TI, and quality of STF, FO, and IF.

	STF	FO	IO
AI	0.63	0.07	0.4
TI	1.28	0.12	0.5
Cholesterol (mg/100 g)	52.21 ± 0.31*	–	50.11 ± 0.57*
AV (mg KOH/g)	0.78 ± 0.03b	0.23 ± 0.03c	0.98 ± 0.02a
POV (mmol/kg)	0.49 ± 0.06a	0.43 ± 0.04b	0.33 ± 0.03c

*Note*: AV and POV were measured according to the Chinese national standard (GB15196‐2015). Values are 3 ± SDs of experiments. The same alphabets indicate the absence of significant differences between the groups (*p* > .05, Tukey's test).

Abbreviations: AI, atherogenic index; AV, acid value; POV, peroxide value; TI, thrombogenic index.

* significantly different from the STF (*p* < .05, unpaired *t*‐test).

## 4 DISCUSSION

ALA is a plant‐derived n‐3 PUFA that has been known to lower the risk of hypertension and coronary heart disease by regulating arachidonic acid metabolism (cyclo‐oxygenase pathway) to decrease inflammatory eicosanoids (Simopoulos, 2004). ALA has also been reported to lower systolic blood pressure, via rescuing SIRT3 dysfunction in endothelial cells, endothelium‐dependent vasodilation in SHR, and angiotensin II‐induced hypertension in mice (Li et al., 2020). Furthermore, in the mouse brain, dietary ALA has been found to attenuate oxidative stress, neuroinflammation, and neuronal apoptosis induced by Cd (Alam et al., 2021). As noted in the introduction above, there is growing interest in plant oils that are rich in ALA, such as FO, perilla oil, and sacha inchi seed oil. This interest has arisen due to the recognition of a dietary shortage of ALA in many countries.

In the present study, the lipase used for the interesterification reaction was selected based on the content of ALA at the sn‐2 position of triglycerides in the reaction product, IF, in the preliminary study. As a result, Lipozyme®RM IM was selected, since it had a stronger activity than the three other lipases examined. Both the sn‐1 and sn‐3 position‐specific lipases, Lipozyme®TL IM and Lipozyme®RM IM, had more or less stronger activities than two other, nonspecific lipases, Lipozyme®CALB and Novozym®435. Lin et al. (2006) reported that the utilization of sn‐1‐ and sn‐3‐specific lipases in interesterification processes resulted in a reduction of triglyceride saturation levels and an increase in the content of UFAs in the final product. Therefore, sn‐1‐ and sn‐3‐specific lipases are potentially suitable for incorporating ALA into the sn‐2 position of triglycerides, compared to nonspecific lipases. Kim and Akoh (2015) have reported that the absorption rate of FAs is notably enhanced when a long‐chain PUFA is positioned at the sn‐2 position of triglycerides. Porsgaard and Høy (2000) reported, using rats, that the intestinal absorption rate of UFAs at the sn‐2 position was higher compared to that of sn‐1 and sn‐3 SFA. The contents (%) of the most of FAs in IF were the averages of those present in STF and FO. OA, LA, and ALA in IF were 33.98 ± 1.34%, 7.92 ± 0.08%, and 23.84 ± 0.65%, respectively. Therefore, IF may be expected to promote ALA absorption.

Generally, most of the FAs in animal fats are OA. Dietary OA is known to have beneficial effects on blood pressure, glucose metabolism, coagulation activity, wound healing, inflammatory diseases, and cancer (Bermudez et al., 2011; Sales‐Campos et al., 2013; Scoditti et al., 2014). It is also found that OA had a neuroprotective effect against oxidative stress and inflammatory response in lipopolysaccharide‐activated murine BV2 microglial cells and might possibly improve Alzheimer's disease (Castellano et al., 2019). OA is one of the major FAs found in STF and its content in STF has been reported to be significantly higher than that in the subcutaneous and perirenal fat of the Sunite sheep (Wang et al., 2019). So, STF appears to have an advantage as a health food at least over the subcutaneous and perirenal fats of Sunite sheep.

During lipase‐catalyzed interesterification between STF and FO at a low temperature (60°C), the molecular bonds of the two fats were rapidly broken and recombined, the content of ALA at the sn‐2 position of IF was increased, and the enzyme did not generate heat for the recombination process of IF at a low temperature, making IF more stable.

STF contains OCSFAs such as pentadecanoic acid and heptadecanoic acid. OCSFAs are found almost exclusively in milk, ruminant meat, and fat, and have been reported to reduce the risk of cardiovascular diseases (Jenkins et al., 2015). Since OCSFAs are not the preferred substrates for FA oxidation, they accumulate in cells where they are used to synthesize very long‐chain FAs needed in the brain (Gotoh et al., 2008; Pfeuffer & Jaudszus, 2016). OCSFAs are also known to prevent breast and prostate cancer (Jenkins et al., 2015; Oskarsson & Ohlsson Andersson, 2016; To et al., 2020; Venn‐Watson et al., 2020). Matejcic et al. (2018), have shown that higher circulating levels of OCSFAs and n‐3 PUFA may be associated with a lower risk of pancreatic cancer. In this respect, therefore, IF intake may be advantageous to living a healthy life.

The sensory properties and ductility of oils are closely related to SFC, and the SFC value of oils at 10°C is <32%, indicating that they have good ductility (Wassell & Young, 2007). In some studies, an SFC of less than 3% at 35°C has been shown to prevent waxy taste (Sarafhana et al., 2016). IF the SFC content is less than 10% at 10°C, and SFC content is 0 at 35°C, indicating that it has good ductility and excellent taste.

The contents in IF of tridecanoic acid and heneicosanoic acid were similar to those in STF. Besides, undecanoic acid was found only in IF. Despite the small quantities of OCSFAs in IF, these FAs may still contribute to the overall merits of IF as a healthy food option. IF contained a higher content of γ‐linolenic acid (GLA) compared to both, STF and FO. The analysis conducted using SIMCA® 14.1 revealed that the contents of undecanoic acid and GLA differentiate IF from both, STF and FO. It is worth noting that undecanoic acid, which was present in IF, was not found in either, STF or FO. This suggests that undecanoic acid might have been formed during the interesterification process. Further research is required to elucidate the causes of these results.

Supplementing both ALA and GLA through the intake of IF may be favorable. GLA has been found to enhance the anti‐inflammatory effects of ALA. However, it is crucial to maintain a proper balance between ALA and LA intake to achieve the optimal effectiveness of this combination. According to the findings of Wang et al. (2021), behenic acid, which is an ultra‐long‐chain SFA, has been identified as a key molecule involved in regulating lifespan and promoting longevity. In this study, the behenic acid content in IF was 167% of that in STF. Moreover, BCFAs and conjugated linoleic acid (CLA) found in STF are expected to have anti‐inflammation or anticancer effects, as well (Ali et al., 2012; Chikwanha et al., 2018; Ip et al., 2003). Based on these findings and existing knowledge, IF containing unique FAs holds potential utility as an edible fat beneficial for health. It may also have practical applications. However, further research is needed to understand fully the reasons behind the increase in undecanoic acid, GLA, and behenic acid in IF. Hou and colleagues (Jiale et al., 2023), in their recent study, reported that the IF developed in this study has the capability to reduce blood low‐density lipoprotein cholesterol (LDL‐C) and to increase high‐density lipoprotein cholesterol (HDL‐C) and liver function in spontaneously hypertensive rats (SHR). By enhancing lipid profiles and liver function, this IF can mitigate symptoms in SHR, leading to an increase in testosterone secretion and a beneficial effect on steroid hormone metabolism. This research highlights the potential health advantages of IF in managing cardiovascular diseases and endocrine imbalances, indicating its possible practical applications in the food and healthcare sectors.

SFC of IF consistently remained lower than that of STF within the temperature range of 0–30°C. Fats and oils generally become harder and less ductile as SFC increases. The change in SFC of IF with temperature reflects its flexibility and softness at room temperature. This behavior is attributable to the melting points of the various FAs bound to the triglyceride molecules present in IF. IF is thought to be well absorbed by the body owing to its complete melting at temperatures above 30°C. Furthermore, it was discovered that IF has lower values of AI, TI, and cholesterol, and greater oxidation stability, compared to STF. These findings indicate that IF may, therefore, offer potential health benefits by having a favorable lipid profile and enhanced oxidation stability.

In summary, IF can be readily obtained by performing ester exchange between STF and FO using Lipozyme®RM IM as the catalyst. This process allows for the production of IF with a unique FA composition and potential health benefits. IF, which exhibits the FA features of both, STF and FO, and possesses easy‐to‐use physicochemical properties, is expected to make significant contributions in several areas. First, it has the potential to serve as a nutritionally beneficial fat, offering a unique FA profile. Additionally, IF can be explored as a new ingredient for the development of novel food and cosmetic products. IF may also help expand the utilization of STF, which is currently not extensively utilized.

## AUTHOR CONTRIBUTIONS

**Jin‐Hua Baoyindugurong:** Conceptualization (equal); funding acquisition (equal); methodology (equal); writing – original draft (equal); writing – review and editing (equal). **Jia‐Le Hou:** Conceptualization (equal); data curation (equal); investigation (equal); methodology (equal); software (equal); writing – review and editing (equal). **Ya‐Nan Ren:** Methodology (equal). **Ya‐Wen Li:** Supervision (equal). **Mailisi Heshuote:** Formal analysis (equal). Investigation (equal). **Hugejiletu:** Investigation (equal). **Naoki Ohara:** Writing – review and editing (equal). **Yukiko Naito:** Writing – review and editing (equal). **Kenjiro Tatematsu:** Writing – review and editing (equal).

## FUNDING INFORMATION

This study was sponsored by the Construction of Academician and Expert Workstation in Inner Mongolia Autonomous Region the Inner Mongolia Autonomous Region Special Fund Project for the Transformation of Scientific and Technological Achievements (2020CG0031 and 2020CG0033); the Inner Mongolia Autonomous Region Department of Finance “Grassland Talents” Project Special Fund The Department of Finance of Inner Mongolia Autonomous Region issued a document [2021] No.333; the Inner Mongolia Autonomous Region Science and Technology Program (2021GG0348); the Inner Mongolia Natural Science Gold Project (2023MS03034).

## CONFLICT OF INTEREST STATEMENT

The authors declare that they have no known competing financial interests or personal relationships that could have appeared to influence the work reported in this paper.

## ETHICS STATEMENT

Animal and human experiments were not conducted in this article, so there are no animal ethics or related documents.

## Data Availability

The data presented in this study are available on request from the corresponding author.
